# Book Reviews, Pamphlets, Exchanges

**Published:** 1903-04

**Authors:** 


### *BOOK REVIEWS, PAMPHLETS, EXCHANGES.*

LEA'S SERIES OF MEDICAL EPITOMES. MANTON'S OBSTETRICS. A Manual of Obstetrics for Students and Practitioners. By W. MANTON, M. D., Adjunct-Professor of Obstetrics and Professor of Clinical Gynecology, Detroit College of Medicine. In one 12mo volume of 265 pages, with 82 illustrations. Cloth $1.00. Lea Brothers & Co., Publishers, Philadelphia and New York, 1903.

The essentials of obstetrics are presented in this book in a manner that is intended fo be as plain and simple as is consistent with a proper elucidation of the subject. At the end of each chapter are lists of questions reviewing the ground gone over. This arrangement is very convenient for the student and much simplifies the work. The small price of the book places it in reach of every medical reader while the arrangement is equally commendable.

---

A TEXT BOOK OF PRACTICAL MEDICINE. By Wm. Gilmam Thompson, M. D., Prof. Medicine in the Cornell University Medical College. Physician to the Presbyterian and Bellevue Hospitals, New York. Second Edition Revised and Enlarged· Lea Bros. & Co., New York and Philadelphia, 1902.

The new edition of this book contains in addition to the commendable features of the first, new material growing out of the progress of medicine in the last two years. There are about fifteen pages of additions to the text. Among infectious diseases, those in the etiology and prevention of which recent work has been done, the articles on dysentery, yellow fever and malaria have been greatly revised and largely rewritten. Articles on Diseases of the Blood and Heart have been to some extent rewritten and additions have been made to the discussion of the diseases of the digestive system.

Clinical Treaties on the Pathology and Theropy of Disorders of the Nutabolism and Nutrision. By Dr. Carl Van Noorden, Frankfort on the Main. Traulsated under the direction of Dr. Boardman Reed, E. B. Treat & Co. Publishers, N. Y.

These treatises by the eminent German Observer and clinican consist of a series of essays dealing with important nutritive disturbances. The first one of the series dealt with obesity the second of treatment of acute nephritis and chronic atrophic kidney and the third membranous catarrh of the intestines. They will read with interest and much profit as they are the direct outcome of the personal views and observations of one who is in position to speak authoritively.

The Iternatioal Medical Annual 1903. Twenty-First year. E. B. Treat & Co. New York.

The contributors to the annual are nearly all English. There are however a few notahle exbeptions and in the list one sees the well-known names of Hare, H. P. Loomis, A. D. Rockwell and G. M. Hammond. A general summary of the work of the year is given and this is followed by the usual sections dealing specifically with the several provinces of medicine. The book is a very acceptable one especially for quick reference. The progress of medicine during the year has been carefully chronicled and every thing of value that has appeared in medical publications has been caught and epitomized. The book is well illustrated.

---

The New York State Hospital for the Care of Crippled and Deformed Children, Tarrytown, has issued its report for the year ended September 30, 1902. During this period thirty-five patients have been treated, of whom ten have been discharged. About one hundred and fifty applications for admission have been made during the year, but owing to the restricted accommodations the larger portion could not be accepted. If the hospital had been equipped with 5,500 beds the surgeon-in-chief says he could have filled them in a month with deserving patients.

